# Computational Analysis of the Morphological Aspects of Triadic Hybridized Magnetic Nanoparticles Suspended in Liquid Streamed in Coaxially Swirled Disks

**DOI:** 10.3390/nano12040671

**Published:** 2022-02-17

**Authors:** Zubair Akbar Qureshi, Sardar Bilal, Imtiaz Ali Shah, Ali Akgül, Rabab Jarrar, Hussein Shanak, Jihad Asad

**Affiliations:** 1 Department of Mathematics, Multan Campus, AIR University, Multan 49501, Pakistan; 2 Department of Mathematics, AIR University, Islamabad 44000, Pakistan; imtiazsha91@gmail.com; 3 Department of Mathematics, Art and Science Faculty, Siirt University, Siirt 56100, Turkey; aliakgul00727@gmail.com; 4 Department of Physics, Faculty of Applied Sciences, Palestine Technical University-Kadoorie, Tulkarm 305, West Bank, Palestine; r.jarrar@ptuk.edu.ps (R.J.); h.shanak@ptuk.edu.ps (H.S.); j.asad@ptuk.edu.ps (J.A.)

**Keywords:** triadic hybridize nanofluid model, heat and mass flux, MHD, morphology effect, computational analysis (shooting technique)

## Abstract

Currently, pagination clearly explains the increase in the thermophysical attributes of viscous hybrid nanofluid flow by varying morphological aspects of inducted triadic magnetic nanoparticles between two coaxially rotating disks. Copper metallic nanoparticles are inserted with three different types of metallic oxide nanoparticles: Al_2_O_3_, Ti_2_O, and Fe_3_O_4_. Single-phase simulation has been designed for the triadic hybrid nanofluids flow. The achieved expressions are transmuted by the obliging transformation technique because of dimensionless ordinary differential equations (ODEs). Runge–Kutta in collaboration with shooting procedure are implemented to achieve the solution of ODEs. The consequences of pertinent variables on associated distributions and related quantities of physical interest are elaborated in detail. It is inferred from the analysis that Cu-Al_2_O_3_ metallic type hybrid nanofluids flow shows significant results as compared with the other hybrid nanoparticles. The injection phenomenon on hybrid nanofluids gives remarkable results regarding shear stress and heat flux with the induction of hybridized metallic nanoparticles. Shape and size factors have also been applied to physical quantities. The morphology of any hybrid nanoparticles is directly proportional to the thermal conductance of nanofluids. Peclet number has a significant effect on the temperature profile.

## 1. Introduction 

Copper is a transitional metal and possesses the ability to accept and donate electrons along with the characteristic to carry out oxidization and reduction. Because of this tendency, it is used as an essential micronutrient for functionality of living organisms and development of enzymes involved in metabolic processes [1,2]. Moreover, copper contributes to the cure of heart disease, diabetes, and obesity by the formation of drugs [3,4,5,6,7]. In recent years, advancement in nanotechnology has generated intent toward findings of innovative procedures for the production of copper particles on the nano scale (1–100 nm). Especially, metallic nanoparticles including copper particles are widely used in medicinal instruments, investigative imaging, drug supply procedure, therapeutics, and cancers cells. In spite of excellent utilizations of copper nanoparticles, some serious dangers of such nanoparticles are continuously elevated as a result of tissue damaging [8,9,10]. For this purpose, hybridization of copper with non-metallic oxides is performed, which makes a new class of working liquids. Extensive experimental research on hybrid nanocomposites can be found, e.g., Turcu et al. [11] and Jana et al. [12]. Furthermore, Devi and Devi [13,14] examined the consequence of hybridized nanoliquid over stretchable configuration and concluded on enhancement in the thermal rate with inclusion of hybridized nanoparticles in base fluid. Farooq et al. [15] analyzed the flow of bio convective cross nanofluid with motile microorganisms in the attendance of radiative energy and melting phenomenon. Anitha et al. [16] evaluated the performance of a double heat exchanger by considering two different hybrid nanofluids composed of water and ethylene as base liquids and TiO_2_-γ-AlOOH nanoparticles. Ebrahimi et al. [17] showed modelling of laminarly convective heat transfer of nanoliquid in an enclosure by implementing the finite element approach. 

Rotating flows is one of the basic frameworks in the dynamics of liquids. The pioneering work in this direction was performed by Karman [18], who developed the formulation of such a problem by forming the Navier–Stokes equation in curvilinear coordinates. Following the work presented by Karman, extensive works on rotational flows have been performed, such as Griffiths [19], who executed the flow mechanism of non-Newtonian liquid over a spinning disk. Some valuable and old literature related to rotational flow problems is encapsulated in [20,21,22,23,24]. Waini et al. [25] examined the influence of hybrid copper and aluminum oxide nanoparticles in heat transfer elevation of water on a rotating disk. Turkyilmazoglu [26] inspected the single-phase flow of nanofluid over a rotated disk by determining Brownian diffusion aspects. Turkyilmazoglu [27] analyzed 3D laminar flow of electrically conducting viscous liquid flowing over a rotating disk. Some recent acquisitions on rotatory flow problems are divulged in [28,29,30,31].

Synthetic characteristics of hybrid nanoparticles along with superior fluidity and stability properties and practical utilization of these composite particles have been raised in different technological applications such as electronic cooling devices, thermal control of vehicles, welding, power systems, lubrication, hydroelectric manufacturing, production of paper and biomedicine, nuclear production, manufacture of spacecraft devices, and many other areas [32,33,34]. Xu et al. [35] explained the unsteady mixed convective flow of a hybrid nanoliquid between spinning disks. The influence of Hall current and magnetic field on hybrid nanoliquid flow between coaxially rotated disks was encapsulated by Nilankush et al. [36]. Dinarvand et al. [37] computationally scrutinized the flow behavior of a hybrid nanofluid over a porous rotating disk with induction of metallic-oxide (ZuO-Au). Khan et al. [38] probed impression of the Hall effect on a hybrid nanoliquid flowing on a spherical surface. Izadi et al. [39] delineated convective heat transfer in a water hybrid nanofluid with induction of multi-wall carbon nanotubes inside an enclosure. Arani et al. [40] depicted the heat and flow characteristics of a laminar water-based nanoliquid in a novel design of a double-layered microchannel heat sink. Safaei et al. [41] presented work on the thermal aspects of functionalized multi-walled carbon nanotubes in nanoliquid flow over a flat plate by performing numerical simulations. Goshayeshi et al. [42] determined the influence of the shape and size of nanoparticles in elevation in the heat transfer rate of a pulsating heat pipe under the influence of magnetic field. An overview about work conducted by a researcher regarding a hybrid nanoliquid over rotating disks is accumulated in [43,44].

Combined evaluation of heat and mass transfer phenomenon has superb applications in chemical and food processing, hydrometallurgy, ceramics manufacturing, polymerization, and so forth. Vajravelu et al. [45] analyzed magnetically effected 3D squeezed flow of the nanoliquid between rotating discs with velocity slip. Das et al. [46] presented mathematical modelling of magnetically influenced squeezed nanoliquid flow between coaxially rotating disks. Heat and mass transfer aspects in MHD squeezed flow with dispersion of nanoparticles by providing slip effects on surface of disk were deliberated by Din et al. [47]. Qayyum et al. [48] studied heat and mass change in nanofluid thermal flux across a spinning disk with a uniform thin layer. Aziz et al. [49] adumbrated heat and mass transport in dissipated and magnetized flow of viscous fluid over a spinning disk. Reddy et al. [50] presented an analysis on enhancement in convective heat and mass transfer with the addition of metallic hybridized metallic nanoparticles in fluid flow over a rotating disk. 

MHD is the study of the fluid flow mechanism under the influence of magnetic field. Magnetized fluid possesses significant applications in different fields, especially in biomedical science like laser beam scanning, drug delivery targeting, manipulation of nanoparticles, MHD base micropump, magnetic rays imaging, and many others. Muhammad et al. [51] probed the flow of viscoelastic liquid under the impact of magnetic field. Uddin et al. [52] discussed the impact of magnetization on nano viscous liquid over a rotating permeable disk. The effects of magnetic field on viscously dissipated hybrid nanofluids by performing numerical simulations were reported by Imran et al. [53]. Khan et al. [54] evaluated the magnetic field effect on flow features of viscous liquid between coaxially rotated disks. Some recent literature surveys regarding the influence of magnetic field on fluid flow problem in multiple computational domains and under the consideration of various physical variables are accumulated like Krishna et al. [55,56] discussed the consequence of magnetic field along with hall and ion slip on second grade rotating fluid on a semi-infinite vertically moving surface. 3D convective heat transfer in micro concentrated annulus generated by non-uniform heat flux at wall in water base nanofluid with induction of Al_2_O_3_ nanoparticles was determined by Davood et al. [57]. Some recent developments on MHD fluid in different computation domains are gathered in [58,59,60,61,62,63,64,65,66,67].

Examination of fluid flow phenomenon in porous orthogonal disks has numerous dedicated utilizations in many advanced technologies, such as lubricants bearing technology, mass and heat exchanger, viscometers, crystal growth, biomechanics, oceanography, and computer storage system. In this geometry, the foremost physical aspect is the injection/suction along with consideration of hybrid nanofluids, which makes this problem more remarkable in view of practical essence. So, the main objective here is to investigate the enhancement in the thermophysical characteristics of water by inserting triadic hybridized nanoparticles in flow between two orthogonally moving permeable disks. After reviewing the aforementioned literature, consideration of triadic nano particles inside fluid domains is not scrutinized yet. Therefore, the prime concern of this pagination is to inspect the behavior of flow concerning profiles like shear stress, velocity, temperature, and mass profile for injection/suction cases with the addition of triadic particles. A solution to the problem at hand is heeded by implementing the shooting technique and a comparison of computed data with published literature is revealed.

## 2. Problem Formulation

**Figure 1:**
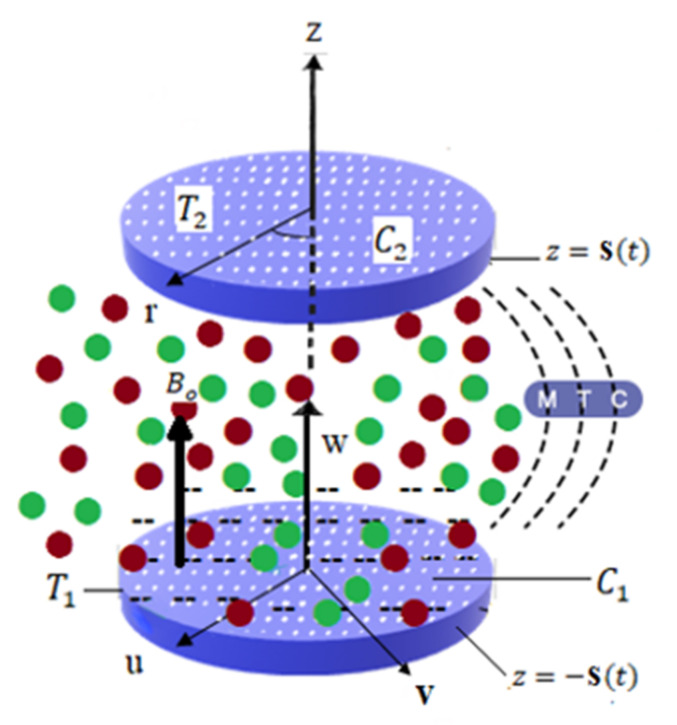
Physical model.

Here, we assume unsteady, laminarly, and 3D viscous liquid over rotating permeable disks in the attendance of externally produced magnetic field. Here, we ignore the Hall current effect and body forces, i.e., induced magnetic field due to the presence of external pressure by providing low magnitude of permeable Reynold number. Single-phase simulation was developed for the problem at hand in the presence of different types of nanoparticles. Permeable disks are located at equal distances from the center and moving up and down with distance 2s(t), along with velocity s’(t). Here, the base fluid is water to support our single-phase simulation of the hybrid nanofluids flow. The triadic type of hybrid composite material was introduced here, in which metallic particles of copper are commuted with different metallic-oxide nanomaterials. It is noted that the temperature and concentration of the lower disk are strictly greater than those of the upper disk, as exhibited in Figure 1. 

The constitutive expressions can be written as follows [60]:

(1) $$\frac{\partial u}{\partial r}+\frac{u}{r}+\frac{\partial w}{\partial z}=0,$$

(2) $$\frac{\partial u}{\partial t}+u\frac{\partial u}{\partial r}+w\frac{\partial u}{\partial z}-\frac{v^{2}}{r}=-\frac{1}{\rho_{hnf}}\frac{\partial p}{\partial r}+\upsilon_{hnf}(\frac{\partial^{2}u}{\partial r^{2}}+\frac{1}{r}\frac{\partial u}{\partial r}-\frac{u}{r^{2}}+\frac{\partial^{2}u}{\partial z^{2}})-\;\frac{\sigma_{e}B_{0}^{2}}{\rho_{hnf}}u,$$

(3) $$\frac{\partial v}{\partial t}+u\frac{\partial v}{\partial r}+w\frac{\partial v}{\partial z}+\frac{uv}{r}=\upsilon_{hnf}(\frac{\partial^{2}v}{\partial r^{2}}+\frac{1}{r}\frac{\partial v}{\partial r}-\frac{v}{r^{2}}+\frac{\partial^{2}v}{\partial z^{2}})-\frac{\sigma_{e}B_{0}^{2}}{\rho_{hnf}}v,$$

(4) $$\frac{\partial w}{\partial t}+u\frac{\partial w}{\partial r}+w\frac{\partial w}{\partial z}=-\frac{1}{\rho_{hnf}}\frac{\partial p}{\partial z}+\upsilon_{hnf}\left(\frac{\partial^{2}w}{\partial r^{2}}+\frac{1}{r}\frac{\partial w}{\partial r}+\frac{\partial^{2}w}{\partial z^{2}}\right)$$

(5) $$\frac{\partial T}{\partial t}+u\frac{\partial T}{\partial r}+w\frac{\partial T}{\partial z}=\alpha_{hnf}\frac{\partial^{2}T}{\partial z^{2}}+\frac{\mu_{hnf}}{{\left(\rho c\right)}_{p_{hnf}}}{\left(\frac{\partial u}{\partial z}\right)}^{2}+\;\frac{\sigma_{e}B_{0}^{2}\;u^{2}}{\;{\left(\rho c_{p}\right)}_{hnf}}$$

(6) $$\frac{\partial C}{\partial t}+u\frac{\partial C}{\partial r}+w\frac{\partial C}{\partial z}=\frac{\partial^{2}C}{\partial z^{2}}$$

(7) $$\upsilon_{hnf}=\frac{\mu_{hnf}}{\rho_{hnf}}\;\mathrm{and}\;\;\;\alpha_{hnf}=\frac{k_{hnf}}{{\left(\rho c_{p}\right)}_{hnf}\;},Pr=\frac{{\left(\mu C_{p}\right)}_{bf}}{k_{bf}}$$

where $\rho_{s}\;\mathrm{and}\;\rho_{f}\;$represent the densities of solid particles, particles and fluid; $\;{\left(c_{p}\right)}_{hnf}\;$shows the heat capacitance of hybrid nanofluids; and $k_{hnf}$ is the thermal conductivity of the hybrid nanofluids.

Uniform mixing of nanoparticles and hosting liquid with negligible slip is presumed in the single-phase approach, which produces thermophysical characteristics that are found by experimental calculation. The accuracy of this approach is predominantly dependent on the accurate prediction of the thermophysical properties of the nanofluids, which are thus presently estimated using the same property correlations utilized in the respective experimental study [51].

### Boundary Condition

The associated boundary conditions are as follows:

$$z_{1}=-s\left(t\right)\;u=0\;v=-\frac{rA_{1}s^{\prime }\left(t\right)}{2s}\;w=-A_{1}s^{\prime }\left(t\right)\;T=T_{1}\;C=C_{1}\;$$

and

(8) $$z_{1}=s\left(t\right)\;u=0\;v=\frac{rA_{1}s^{\prime }\left(t\right)}{2s}\;\;w=A_{1}s^{\prime }\left(t\right)\;T=T_{2}\;C=C_{2}$$

For elimination of the pressure term, the following similarity variables are utilized:

$$\eta =\frac{z_{1}}{s}\;u=-\frac{r\upsilon_{f}}{s^{2}}F_{\eta }\left(\eta ,t\right)\;v=\frac{r\upsilon_{f}}{s^{2}}G\left(\eta ,t\right)\;w=\frac{2\upsilon_{f}}{s}F\left(\eta ,t\right)\;\;\;\;\;\;\;\theta =\frac{T-T_{2}}{T_{1}-T_{2}}\;\;,\;\chi \left(\eta \right)=\frac{C-C_{2}}{C_{1}-C_{2}},$$

and

(9) $$\frac{\upsilon_{hnf}}{\upsilon_{f}}F_{\eta \eta \eta \eta }+\alpha (3F_{\eta \eta }+\eta F_{\eta \eta \eta })-2FF_{\eta \eta \eta }-\frac{s^{2}}{\upsilon_{f}}F_{\eta \eta t}+2GG_{\eta }-\frac{\rho_{f}}{\rho_{hnf}}MF_{\eta \eta }=0,$$

(10) $$\frac{\upsilon_{hnf}}{\upsilon_{f}}G_{\eta \eta }+\alpha (2G+\eta F_{\eta })+2GF_{\eta }-\frac{s^{2}}{\upsilon_{f}}G_{t}-2FG_{\eta }-\frac{\rho_{f}}{\rho_{hnf}}\mathrm{MG}=0,$$

(11) $$\theta_{\eta \eta }+\frac{\upsilon_{f}}{\alpha_{hnf}}(\alpha \eta -2F)\theta_{\eta }+[{\left(1-\left(\varphi_{1}+\varphi_{2}\right)\right)}^{-2.5}F_{\eta \eta }{}^{2}+MF_{\eta }{}^{2}]E_{c}P_{r}\frac{\kappa_{f}}{\kappa_{hnf}}-\frac{k^{2}}{\alpha_{nf}}\theta_{t}=0,$$

(12) $$D\chi^{\prime \prime }+\upsilon_{f}(\alpha \eta -2F)\chi^{\prime }-k^{2}\chi_{t}=0,$$

Boundary conditions in dimensionless form are as follows:

$$\eta =-1,\;F=-Re,F_{\eta }=0,\theta =1,\chi =1,$$

and

$$\eta =1,\;F=Re,F_{\eta }=0,\theta =0,\;\chi =0$$

where α = $\frac{ss^{\prime }\left(t\right)}{\upsilon_{f}}$ stands for wall expansion ratio, Re = $\frac{A_{1}ss^{\prime }\left(t\right)}{2\upsilon_{f}}$ stands for permeable Reynold number, $Pr=\frac{{\left(\mu c_{p}\right)}_{f}}{k_{f}}$ stands for the Prandtl number, $Ec=\frac{U^{2}}{\left(T_{1}-T_{2}\right){\left(c_{p}\right)}_{f}}$ stands for Eckert number, $\;P_{e}=Re*P_{r}$ is the Peclet number, Sc = $\frac{\upsilon_{f}}{D}$ stands for Schmidt number, and M =$\;\frac{\sigma_{e}B_{0}^{2}s^{2}}{\mu_{f}}\;$stands for magnetic parameter referred to [60,61].

Finally, we set F = f Re, G = g Re by following Majdalani et al. [61] when α is a constant.

f = f (η) and *θ* = *θ*(η), which leads to $\theta_{t}$ = 0,$\;g_{\eta t}$ = 0, $f_{\eta \eta t}$ = 0, and $\chi_{t}=0$. Thus, we have the following equations:

(13) $$\frac{\upsilon_{hnf}}{\upsilon_{f}}f_{\eta \eta \eta \eta }+\alpha (3f_{\eta \eta }+\eta f_{\eta \eta \eta })-2ff_{\eta \eta \eta }+2Regg_{\eta }-\frac{\rho_{f}}{\rho_{hnf}}Mf_{\eta \eta }=0$$

(14) $$\frac{\upsilon_{hnf}}{\upsilon_{f}}g_{\eta \eta }+\alpha (2g+\eta g_{\eta })+2Re\left(gf_{\eta }-fg_{\eta }\right)-\frac{\rho_{f}}{\rho_{hnf}}Mg=0$$

(15) $$\theta_{\eta \eta }+\frac{\upsilon_{f}}{\alpha_{hnf}}Pr(\alpha \eta -2Ref)\theta_{\eta }+[{\left(1-\left(\varphi_{1}+\varphi_{2}\right)\right)}^{-2.5}f_{\eta \eta }{}^{2}+Mf_{\eta }{}^{2}]ReE_{c}P_{e}\frac{\kappa_{f}}{\kappa_{hnf}}=0$$

(16) $$\chi^{\prime \prime }+Sc(\alpha \eta -2fRe)\chi^{\prime }=0$$

$$\eta =-1;\;f=-1,\;f_{\eta }=0,\theta =1,\;\chi =1$$

and

(17) $$\eta =1;\;f=1,\;f_{\eta }=0,\theta =0\;\chi =0.$$

## 3. Practical and Engineering Interest

### 3.1. Skin Friction Coefficients

The $C_{f1}\;$ and $C_{f-1}$ are expressed as follows:

(18) $$\begin{array}{l} C_{f-1}=\frac{\xi_{w}{|}_{\eta \;=-1}}{\rho_{bf}{\left(s^{\prime }A_{1}\right)}^{2}}\\ =\frac{(1+0.1008({\left(\varphi_{1}\right)}^{0.69574}\left(dp_{1}{)}^{0.44708}+{\left(\varphi_{2}\right)}^{0.69574}{\left(dp_{2}\right)}^{0.44708}\right)}{Re_{r}}\sqrt{{\left(f^{\prime \prime }\left(-1\right)\right)}^{2}+{\left(g^{\prime }\left(-1\right)\right)}^{2}}\\ C_{f1}=\frac{\xi_{w}{|}_{\eta \;=1}}{\rho_{bf}{\left(s^{\prime }A_{1}\right)}^{2}}=\frac{(1+0.1008({\left(\varphi_{1}\right)}^{0.69574}\left(dp_{1}{)}^{0.44708}+{\left(\varphi_{2}\right)}^{0.69574}{\left(dp_{2}\right)}^{0.44708}\right)}{Re_{r}}\sqrt{{\left(f^{\prime \prime }\left(1\right)\right)}^{2}+{\left(g^{\prime }\left(1\right)\right)}^{2}} \end{array}$$

where $\xi_{w}$ stand for total shear stress and $Re_{r}=4\left(\frac{s}{r}\right)\left(\frac{1}{{\left(Re\right)}^{2}}\right)$ stands for local the Reynold number.

$$\xi_{zr}=\mu_{hnf}(\frac{\partial u}{\partial z}){|}_{\eta \;=-1}=\mu_{bf}(1+0.1008({\left(\varphi_{1}\right)}^{0.69574}\left(dp_{1}{)}^{0.44708}+{\left(\varphi_{2}\right)}^{0.69574}{\left(dp_{2}\right)}^{0.44708}\right)\left(\frac{r\upsilon_{f}}{s^{3}}\right)f^{\prime \prime }\left(-1\right)$$

$$\xi_{\theta z}=\mu_{hnf}(\frac{\partial v}{\partial z}){|}_{\eta \;=-1}=\mu_{bf}(1+0.1008({\left(\varphi_{1}\right)}^{0.69574}\left(dp_{1}{)}^{0.44708}+{\left(\varphi_{2}\right)}^{0.69574}{\left(dp_{2}\right)}^{0.44708}\right)\left(\frac{r\upsilon_{f}}{s^{3}}\right)g^{\prime }\left(-1\right)$$

### 3.2. Nusselt Numbers

$Nu_{z-1}$ and $Nu_{z1}$ are given as

(19) $$\begin{array}{c} Nu_{z-1}=\frac{se_{z}}{\kappa_{f}\left(T_{1}-T_{2}\right)}{|}_{\eta \;=-1}=-\frac{k_{hnf}}{k_{f}\;}\theta^{\prime }\left(-1\right)\\ Nu_{z1}=\frac{se_{z}}{\kappa_{f}\left(T_{1}-T_{2}\right)}{|}_{\eta \;=1}=-\frac{k_{hnf}}{k_{f}\;}\theta^{\prime }\left(1\right) \end{array}$$

Here, heat flux is denoted as $s_{z}$, which is as follows:

$$e_{z}{|}_{\eta \;=-1}=-k_{hnf}\left(\frac{\partial T}{\partial z}\right){|}_{\eta \;=-1}=-\frac{\left(T_{1}-T_{2}\right)}{s}k_{hnf}\theta^{\prime }\left(-1\right)$$

$$e_{z}{|}_{\eta \;=1}=-k_{hnf}\left(\frac{\partial T}{\partial z}\right){|}_{\eta \;=1}=-\frac{\left(T_{1}-T_{2}\right)}{s}k_{hnf}\theta^{\prime }\left(1\right)$$

where$\;Re=\frac{A_{1}ss^{\prime }\left(t\right)}{2\upsilon_{f}}$. 

### 3.3. Sherwood Number

Sherwood number is the ratio of convectional mass transfer and diffusion mass transfer. The mass transfer rate (Sherwood number) $Sh{|}_{\eta \;=-1}$ and $Sh{|}_{\eta \;=1}$ at the lower and upper disk have the following mathematical expression:

(20) $$\begin{array}{l} Sh{|}_{\eta \;=-1}=\frac{kq_{z}}{D_{hnf}\left(C_{1}-C_{2}\right)}{|}_{\eta \;=-1}=-\chi^{\prime }\left(-1\right)\\ Sh{|}_{\eta \;=1}=\frac{kq_{z}}{D_{hnf}\left(C_{1}-C_{2}\right)}{|}_{\eta \;=1}=-\chi^{\prime }\left(1\right) \end{array}$$

where

$$q_{z}{|}_{\eta \;=-1}=-D_{hnf}\left(\frac{\partial C}{\partial z}\right){|}_{\eta \;=-1}=-D_{hnf}\frac{\left(C_{1}-C_{2}\right)}{k}\;\chi^{\prime }\left(-1\right)$$

$$q_{z}{|}_{\eta \;=1}=-D_{hnf}\left(\frac{\partial C}{\partial z}\right){|}_{\eta \;=1}=-D_{hnf}\frac{\left(C_{1}-C_{2}\right)}{k}\chi^{\prime }\left(1\right)$$

where$\;Re=\frac{A_{1}ss^{\prime }\left(t\right)}{2\upsilon_{f}}$.

### 3.4. Thermophysical Properties

PDE Equations (13)–(16) have appropriate thermophysical properties:

(21) $$\begin{array}{c} \left(\frac{(1+0.1008({\left(\varphi_{1}\right)}^{0.69574}\left(dp_{1}{)}^{0.44708}+{\left(\varphi_{2}\right)}^{0.69574}{\left(dp_{2}\right)}^{0.44708}\right)}{\left(\left(1-\left(\varphi_{1}+\varphi_{2}\right)\right)+(\varphi_{1})\left(\frac{\rho_{s_{1}}}{\rho_{bf}}\right)+\left(\varphi_{2}\right)\left(\frac{\rho_{s_{2}}}{\rho_{bf}}\right)\right)}\right)f^{\prime \prime \prime \prime }\left[\eta \right]-\alpha (3f^{\prime \prime }\left[\eta \right]+\eta f^{\prime \prime \prime }\left[\eta \right])-\\ 2Ref\left[\eta \right]f^{\prime \prime \prime }\left[\eta \right]-\left(\frac{1}{\left(\left(1-\varphi_{1}-\varphi_{2}\right)+\varphi_{1}\left(\frac{\rho_{s_{1}}}{\rho_{bf}}\right)+\varphi_{2}\left(\frac{\rho_{s_{2}}}{\rho_{bf}}\right)\right)}\right)\;M\;f^{\prime \prime }[\eta ]=0 \end{array}$$

(22) $$\begin{array}{c} \left(\frac{(1+0.1008({\left(\varphi_{1}\right)}^{0.69574}\left(dp_{1}{)}^{0.44708}+{\left(\varphi_{2}\right)}^{0.69574}{\left(dp_{2}\right)}^{0.44708}\right)}{\left(\left(1-\left(\varphi_{1}+\varphi_{2}\right)\right)+(\varphi_{1})\left(\frac{\rho_{s_{1}}}{\rho_{bf}}\right)+\left(\varphi_{2}\right)\left(\frac{\rho_{s_{2}}}{\rho_{bf}}\right)\right)}\right)g^{\prime \prime }\left[\eta \right]+\alpha (2g\left[\eta \right]+\eta g^{\prime }\left[\eta \right])+\\ 2Re\left(g\left[\eta \right]f^{\prime }\left[\eta \right]-f\left[\eta \right]g^{\prime }\left[\eta \right]\right)-\left(\frac{1}{\left(\left(1-\varphi_{1}-\varphi_{2}\right)+\varphi_{1}\left(\frac{\rho_{s_{1}}}{\rho_{bf}}\right)+\varphi_{2}\left(\frac{\rho_{s_{2}}}{\rho_{bf}}\right)\right)}\right)Mg\left[\eta \right]=0 \end{array}$$

$$\begin{array}{l} \theta^{\prime \prime }\left[\eta \right]\\ +(\left(1-\left(\varphi_{1}+\varphi_{2}\right)\right)+\left(\varphi_{1}\right)\left(\frac{\rho_{cps_{1}}}{\rho_{cpbf}}\right)\\ +\left(\varphi_{2}\right)\left(\frac{\rho_{cps_{2}}}{\rho_{cpbf}}\right))\left(\frac{k_{s2}+\left(N-1\right)k_{mbf}+\varphi_{2}\left(k_{mbf}-k_{s2}\right)}{k_{s2}+\left(N-1\right)k_{mbf}-\left(N-1\right)\varphi_{2}\left(k_{mbf}-k_{s2}\right)}\right)\left(\frac{k_{s1}+\left(N-1\right)k_{bf}+\varphi_{1}\left(k_{bf}-k_{s1}\right)}{k_{s1}+\left(N-1\right)k_{bf}-\left(N-1\right)\varphi_{1}\left(k_{bf}-k_{s1}\right)}\right) \end{array}$$

(23) $$\begin{array}{c} Pr(\alpha \eta -2Ref\left[\eta \right])\theta^{\prime }\left[\eta \right]+[{\left(1-\left(\varphi_{1}+\varphi_{2}\right)\right)}^{-2.5}f_{\eta \eta }{}^{2}+\\ Mf_{\eta }{}^{2}]ReE_{c}P_{e}\left(\frac{k_{s2}+\left(N-1\right)k_{mbf}+\varphi_{2}\left(k_{mbf}-k_{s2}\right)}{k_{s2}+\left(N-1\right)k_{mbf}-\left(N-1\right)\varphi_{2}\left(k_{mbf}-k_{s2}\right)}\right)\left(\frac{k_{s1}+\left(N-1\right)k_{bf}+\varphi_{1}\left(k_{bf}-k_{s1}\right)}{k_{s1}+\left(N-1\right)k_{bf}-\left(N-1\right)\varphi_{1}\left(k_{bf}-k_{s1}\right)}\right)=0 \end{array}$$

(24) χ″η+Scαη−2Refηχ′η=0

(25) $$H_{1}=\left(\frac{(1+0.1008({\left(\varphi_{1}\right)}^{0.69574}\left(dp_{1}{)}^{0.44708}+{\left(\varphi_{2}\right)}^{0.69574}{\left(dp_{2}\right)}^{0.44708}\right)}{\left(\left(1-\left(\varphi_{1}+\varphi_{2}\right)\right)+(\varphi_{1})\left(\frac{\rho_{s_{1}}}{\rho_{bf}}\right)+\left(\varphi_{2}\right)\left(\frac{\rho_{s_{2}}}{\rho_{bf}}\right)\right)}\right)$$

(26) $$H_{2}=\left(\frac{1}{\left(\left(1-\varphi_{1}-\varphi_{2}\right)+\varphi_{1}\left(\frac{\rho_{s_{1}}}{\rho_{bf}}\right)+\varphi_{2}\left(\frac{\rho_{s_{2}}}{\rho_{bf}}\right)\right)}\right)$$

(27) $$H_{3}=\left(\left(1-\left(\varphi_{1}+\varphi_{2}\right)\right)+\left(\varphi_{1}\right)\left(\frac{\rho_{cps_{1}}}{\rho_{cpbf}}\right)+(\varphi_{2})\left(\frac{\rho_{cps_{2}}}{\rho_{cpbf}}\right)\right)$$

(28) $$D_{1}=\left(\frac{k_{s2}+\left(N-1\right)k_{mbf}+\varphi_{2}\left(k_{mbf}-k_{s2}\right)}{k_{s2}+\left(N-1\right)k_{mbf}-\left(N-1\right)\varphi_{2}\left(k_{mbf}-k_{s2}\right)}\right)$$

(29) $$D_{2}=\left(\frac{k_{s1}+\left(N-1\right)k_{bf}+\varphi_{1}\left(k_{bf}-k_{s1}\right)}{k_{s1}+\left(N-1\right)k_{bf}-\left(N-1\right)\varphi_{1}\left(k_{bf}-k_{s1}\right)}\right)$$

(30) $$\omega =D_{1}D_{2}.$$

Putting values of (25)–(30) in Equations (21)–(24), the final result is

(31) $$H_{1}f^{\prime \prime \prime \prime }\left[\eta \right]-\alpha (3f^{\prime \prime }\left[\eta \right]+\eta f^{\prime \prime \prime }\left[\eta \right])-2Ref\left[\eta \right]f^{\prime \prime }\left[\eta \right]-H_{2}M\;f^{\prime \prime }[\eta ]=0$$

(32) $$H_{1}g^{\prime \prime }\left[\eta \right]+\alpha (2g\left[\eta \right]+\eta g^{\prime }\left[\eta \right])+2Re\left(g\left[\eta \right]f^{\prime }\left[\eta \right]-f\left[\eta \right]g^{\prime }\left[\eta \right]\right)-H_{2}Mg\left[\eta \right]=0$$

(33) $$\theta^{\prime \prime }\left[\eta \right]+H_{3}\omega Pr(\alpha \eta -2Ref\left[\eta \right])\theta^{\prime }\left[\eta \right]+\omega [{\left(1-\left(\varphi_{1}+\varphi_{2}\right)\right)}^{-2.5}f_{\eta \eta }{}^{2}+Mf_{\eta }{}^{2}]ReE_{c}P_{e}=0$$

(34) χ″η+Scαη−2Refηχ′η=0

### 3.5. Solution Procedure

This segment is presented for the discussion of the implemented numerical scheme and steps involved during the simulations. For this purpose, firstly, Equations (13)–(16) with boundary conditions along with effective thermophysical properties are solved numerically by implementing the numerical scheme renowned as RK 4th order in conjunction with the shooting method. To achieve the solution from these procedures, initially, numerical values are choose carefully to accomplish the desired level of accuracy. Owing to low computation cost and memory loss and provision of accurate and consistent results in less time, Runge–Kutta and shooting methods are applied.

## 4. Result and Discussion

In the present section, results on both the graphical and tabular form against different parameters such as the expansion/contraction ratio parameter $\left(\alpha \right)$, permeable Reynold parameter (Re), magnetic parameter (M), Prandtl number (Pr), diameter/size of the nanoparticles $\left(dp_{1}\right)$ and $(dp_{2}$), shape factor of the nanoparticles (N), Peclet number (Pe), and Eckert number (Ec), as well as nanoparticle volume fraction $\left(\varphi \right)$ on velocity, temperature and mass distributions, shear stress, and heat and mass transfer rate, are examined thoroughly. 

Table 1 and Table 2 present the thermophysical properties of (HNFDs) and base fluid with different types of nanoparticles (NPs). Table 3 shows the effect of diameter, volume fraction, and Reynold number on shear stresses by considering three different types of compositions for hybridize NPs, as well as shear stress showing an increasing pattern as compared with tensional stress with an injection factor. Similarly, behavior is observed for the variation in the size of metallic oxides for all types of nanoparticles (Al_2_O_3_, TiO_2_, and Fe_3_O_4_) compared with copper. Nanoparticles have unique features as compared with bulk material of the same structure. The most common properties of the nanoparticles, for example, can be easily rehabilitated by varying their size and shape. Copper nanomaterials have high thermal conductivity as well as electrical conductivity. The most common shape of copper is round visibility, such as black powder. On the other hand, metal oxide nanoparticles are a very important technological material and have many industrial applications.

**Figure 2:**
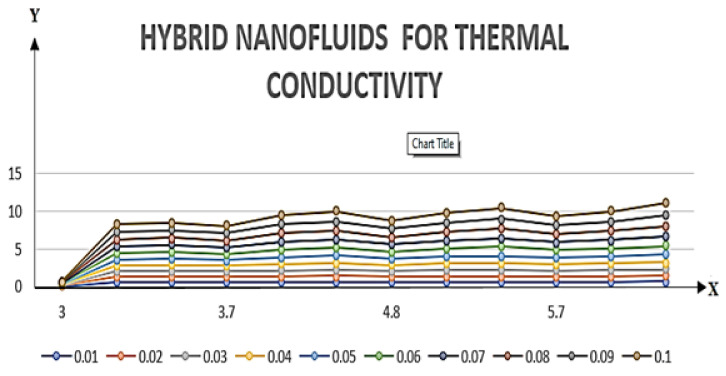
The influence of the scale of hybrid NPs under the thermal conductivity coloring umbrella. Reprinted from [68].

**Figure 3:**
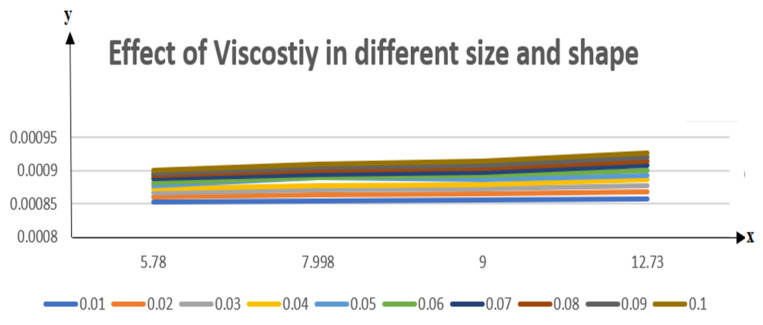
The influence of the scale of hybrid NPs under the viscosity coloring umbrella. Reprinted from [68].

**Figure 4:**
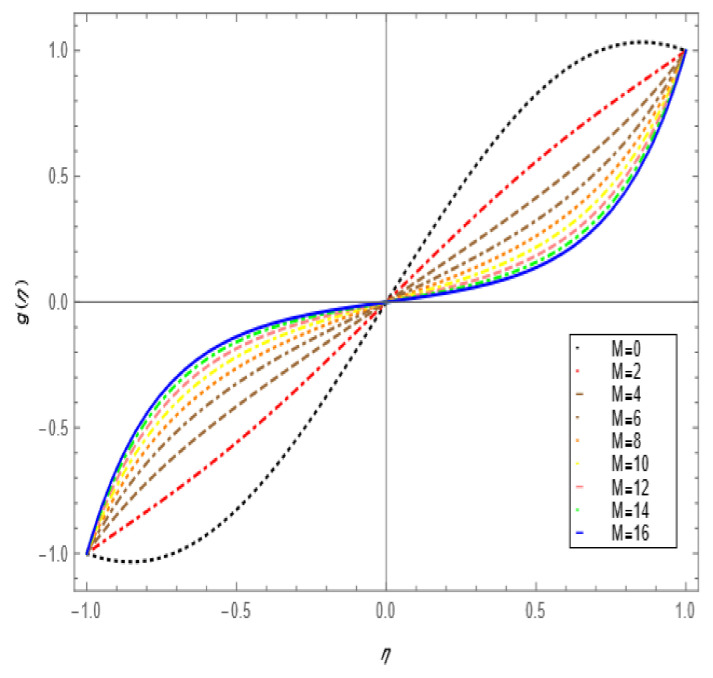
Tangential velocity profile effect for magnetic parameter for =1$,\;Re=1,\;\varphi_{1}=\varphi_{2}=0.01,\;\;Ec=0.00068,\;Pr=6.2$.

**Figure 5:**
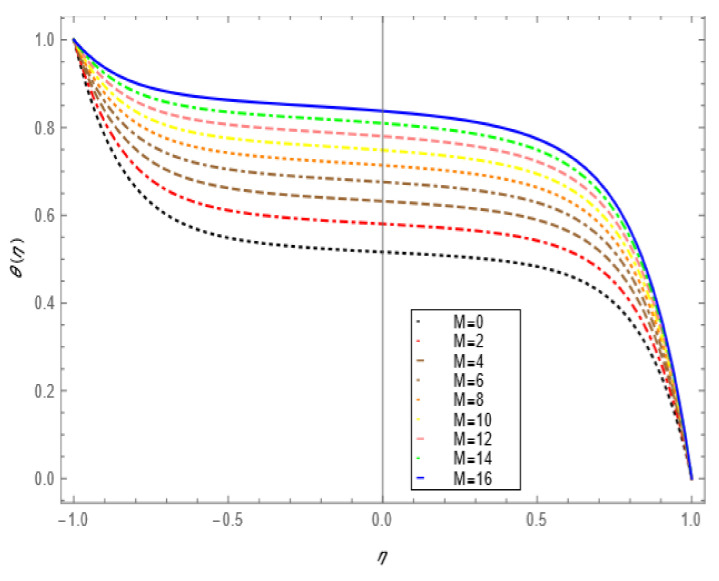
Temperature profile effect for magnetic parameter for =1,$\;Re=1,\;\varphi_{1}=\varphi_{2}=0.01,\;Ec=0.00068,\;Pr=6.2$.

**Figure 6:**
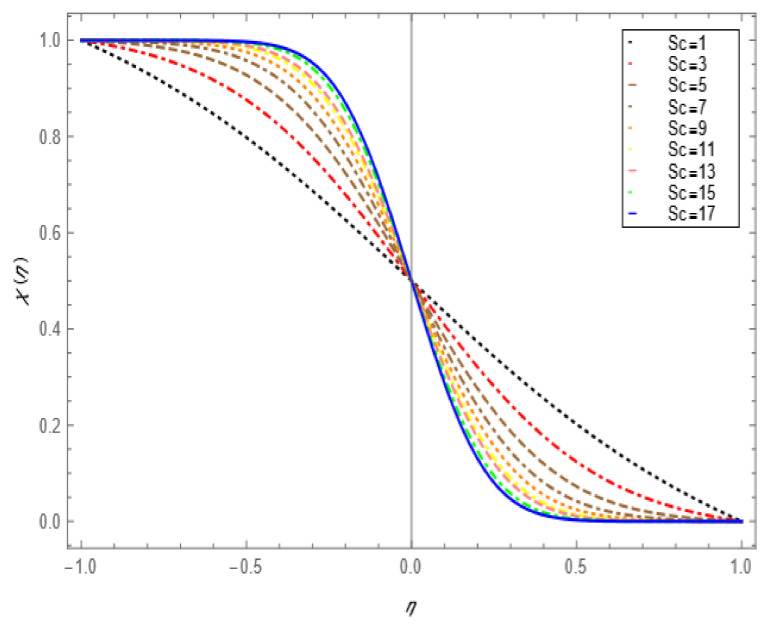
Concentration profile effect for Schmidt number for $=-1,Re=-1,\;\varphi_{1}=\varphi_{2}=0.03,\;Ec=0.00068,\;Pr=6.2,M=1$.

**Figure 7:**
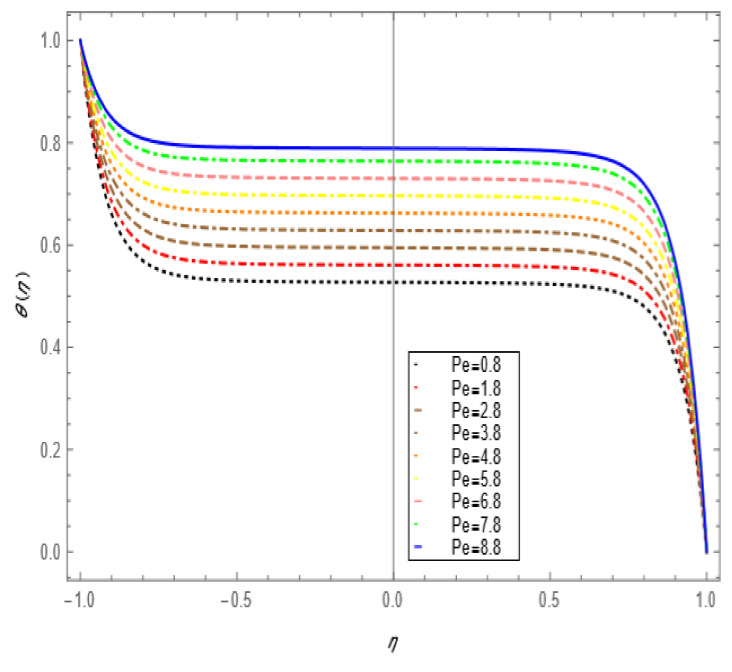
Temperature profile effect size for $=0.1,Re=1.5,\;M=5,\;\varphi_{1}=\varphi_{2}=0.05,\;Ec=0.00068,\;Pr=6.2$.

If we increase the nanoparticle level fraction of copper from 1% to 4%, then shear stress is an increasing function; a similar trend is also observed for metallic oxide nanoparticle fraction. The injection phenomenon is very important for biomedical sciences [46]. When increasing the numerical values of injection number in Table 3, both shear and tensional stresses show an increasing pattern. Table 4 calculates the Nusselt number with different shape factors for hybridizing nanofluids (Cu-TiO_2_/H_2_O, Cu-Fe_3_O_4_/H_2_O, Cu-Al_2_O_3_/H_2_O). If we increase the values of nanoparticles volume fraction,$\;\varphi_{1}$ and $\varphi_{2}$, then the shape factor of (Cu-Al_2_O_3_/H_2_O) shows better performance than the others. Table 5 demonstrates the effect of the permeable Reynold number Re, expansion ratio α, and Sc on Sherwood number of Cu-Al_2_O_3_/H_2_O. Sherwood number has significant results for the suction case, as compared with injection, when α > 0. An opposite trend is observed for contracting and expanding cases when Re < 0. If we increase the numerical values of S, then there is a very significant effect on the Sherwood number. Table 6 displays the effect of different parameters on the heat transfer rate at the lower porous disk. Increases in the Pr and Re numbers significantly enhance the heat transfer rate at the lower porous disk, while, on the other hand, N(shape factor) and M (MHD) have a small effect on the heat transfer rate. The Prandtl number Pr, in our problem, the relative importance of the fluid’s viscosity and thermal conductivity, appears to raise the actual fluid temperature. Table 7 represents the influence of the magnetic parameter M > 0 on the shear stress, tensional stress, and heat transfer rate, and they are all gradually enhanced in the presence of 2% hybrid nanoparticles with the injection case too. In Figure 2, we examine the effect of four types of shape factors on hybrid nanofluid flow associated with a different numerical range of $\varphi_{1}$ and $\varphi_{2}$. All shape factors lie on the x-axis and thermal conductivity varies on the y-axis. A high thermal conductivity value is achieved at 5.7 when the nanoparticle volume fraction is at 1. Thermal conductivity is an increasing function of the shape factor with the nanoparticle volume fraction. We can say that morphology is directly proportional to the thermal conductivity of any nanofluids. By taking equal numerical values of $\varphi_{1}$ and $\varphi_{2},$ in Figure 3, with a viscosity of base fluids and diameter of all nanoparticles along with different numerical values of morphology (spherical, bricks, cylindrical, and platelets), the X-axis denotes the size factor and the y-axis represents the effective viscosity. Furthermore, the effect of viscosity is very high for a size factor of 12.73 when the nanoparticle volume fraction level is 1%. We also observed that platelet-shaped nanocomposites show a better performance on heat and mass transferability as compared with the other shapes of nanoparticles. Figure 4 and Figure 5 show the effect of a magnetic parameter in the tangent velocity profile and the temperature profile influence of fixed value  α=1,Re=1,Pr=6.2,Ec=0.00068. By increasing the numerical values of the magnetic parameter M, the momentum boundary layer thickness decreases from both porous walls in the presence of $\varphi_{1}=$ $\varphi_{2}$ = 1%. The physically generated Lorentz force by amplification of the magnetic field generates a resistance to flow and decreases the momentum boundary layer thickness, which is why heat is the main source of heat production. The conclusion is suitable for the fact that the magnetic field implemented is a resistance force that plays a crucial role in decelerating and directing fluid flow. Figure 5 shows the increasing behavior of the temperature profile from the center and covers the whole domain. Figure 6 is drawn to show the nature of the Schmidt number concerning the concentration profiles $\chi \left(\eta \right)$. The Schmidt number is, theoretically, the conceptual interaction of momentum and mass diffusivity. Owing to the value of the Schmidt number, the diffusivity increases as a function of the decline in fluid concentration. The value of the Schmidt number is inversely proportional to the diffusivity of the Brownian movement. The greater diffusivity of Brownian corresponds to lower concentration profiles of $\;\chi \;\left(\eta \right)$. With the increase in the value of the Schmidt number, concentration boundary later thickness is an increasing function of Sc in the presence of $\varphi_{1}=\varphi_{2}$ = 3%. Figure 7 represents the temperature profile with various values of the Peclet number. With the increase in the value of $P_{e}$, the flow of heat transfer enhancement is significantly increased from both porous disks in the presence of $\varphi_{1}=$ $\varphi_{2}$ = 5%. Physically, the product of Reynold number and Prandtl number is equal to Peclet number which tends to reduce flow velocity in downstream directions and the current factors tend to be one-way properties. In the common perception, it is expected that particles made from high thermal conductivity material should impose high thermal conductivity on nanofluids, but this is not necessary; on the other hand, a famous scientist, Lee et al. [52], conducted experiments with AL_2_O_3_ and CuO as hybrid nanofluids and reported that, though AL_2_O_3_ material had a higher thermal conductivity than CuO, CuO as a nanofluid possesses higher thermal conductivity. The investigator claimed reason that AL_2_O_3_ nanoparticles form a larger nanocluster than CuO nanoparticles in the base fluid water. Hence, some other factor may also be involved.

## 5. Conclusions

We explore the three-dimensional triadic hybrid nanofluid flow behavior with heat and mass transfer aspects in this manuscript, as well as the Newtonian fluid flow through orthogonal porous disks with MHD effects. Metallic and metallic-oxide nanoparticles with morphology effect are considered here. The most important findings are as follows.

Thermal conductivity and viscosity intensity are the highest in HNFD3 with platelet nanoparticles, followed by spherical, brick, and cylindrical nanoparticles, respectively.

By increasing the values magnetic field M, expansion ratio α, and Peclet Pe, there is an improvement in the rate of heat transfer on the porous disk.

- With an increase in the values of volume fraction, the Nusselt number has the largest effect on HNF3 with platelet nanoparticles.
- The momentum boundary layer is gradually increased if we increase the values of permeable Reynold number Re, the diameter of nanoparticles dp_1_ and dp_2_, and magnetic parameter M.
- If the volume fraction $\varphi_{1}$ and $\varphi_{2}$, the diameter of nanoparticles dp_1_ and dp_2_, and chemical reaction of Reynold number Re values are greater than zero, then the rate of shear stress and tensional stress is enhanced.
- By raising the Schmidt number Sc and chemical reaction Reynold number Re, the rate of mass transfer on the porous disk is enhanced.

## Figures and Tables

**Table 1:** Thermophysical properties of HNFDs [62,63,64,65,66].

Properties	(HNFDs)
Density (ρ)	$\rho_{hnf}=\varphi_{1}\rho_{s_{1}}+\varphi_{2}\rho_{s_{\;2}}+\left(1-\varphi_{1}-\varphi_{2}\right)\;\rho_{bf}$
Viscosity (μ)	$\mu_{hnf}=\mu_{bf}(1+0.1008\;{\left((\varphi_{1}\right)}^{0.69574}{\left(dp_{1}\right)}^{0.44708}$$+\;{\left(\varphi_{2}\right)}^{0.69574}{\left(\;dp_{2}\right)}^{0.44708}$))
Heat Capacity $(\rho C_{P}$)	${\left(\rho c_{p}\right)}_{hnf}=\varphi_{1}{\left(\rho c_{p}\right)}_{s_{1}}+\varphi_{2}{\left(\rho c_{p}\right)}_{s_{2}}+\left(1-\varphi_{1}-\varphi_{2}\right)\;{\left(\rho c_{p}\right)}_{bf}$
Thermal Conductivity (K)	$k_{hnf}$$=\left(\frac{k_{s2}+\left(N-1\right)k_{mbf}+\varphi_{2}\left(k_{mbf}-k_{s2}\right)}{k_{s2}+\left(N-1\right)k_{mbf}-\left(N-1\right)\varphi_{2}\left(k_{mbf}-k_{s2}\right)}\right)k_{bf}$ where $k_{bf}$$=\left(\frac{k_{s1}+\left(N-1\right)k_{bf}+\varphi_{1}\left(k_{bf}-k_{s1}\right)}{k_{s1}+\left(N-1\right)k_{bf}-\left(N-1\right)\varphi_{1}\left(k_{bf}-k_{s1}\right)}\right)k_{f}$

**Table 2:** Properties of base fluids and NPs [67].

Base Fluid/NP’s	ρ( $\mathrm{kg}m^{-3})$	$C_{p}$ $\left(J\;kg^{-1}k^{-1}\right)$	$\kappa \left(wm^{-1}k^{-1}\right)$
H_2_O	997.1	4179	0.613
Cu	8933	385	401
Al_2_O_3_	3970	765	40
Fe_3_O_4_	5180	670	9.7
TiO_2_	4250	686.2	8.9538

**Table 3:** Different parameters of the effect in shear stress and tensional stress.

					**Cu-Al_2_O_3_/H_2_O**		**Cu-TiO_2_/H_2_O**		**Cu-Fe_3_O_4_/H_2_O**	
$dp_{1}$	$dp_{2}$	$\varphi_{1}$	$\varphi_{2}$	Re	$f^{\prime \prime }\left(-1\right)$	$g^{\prime }\left(-1\right)$	$f^{\prime \prime }\left(-1\right)$	$g^{\prime }\left(-1\right)$	$f^{\prime \prime }\left(-1\right)$	$g^{\prime }\left(-1\right)$
1	1	0.01	0.01	0.2	2.0587	−1.7628	2.0593	−1.7629	2.0615	−1.7633
2					2.0604	−1.7545	2.0611	−1.7547	2.0632	−1.7551
3					2.0616	−1.7484	2.0623	−1.7485	2.0644	−1.7489
4					2.0627	−1.7434	2.0634	−1.7435	2.0654	−1.7439
1	2				2.0604	−1.7546	2.0611	−1.7547	2.0632	−1.7551
	3				2.0616	−1.7484	2.0623	−1.7485	2.0645	−1.7489
	4				2.0627	−1.7434	2.0633	−1.7435	2.0656	−1.7439
	1	0.02			2.0799	−1.7519	2.0806	−1.7521	2.0828	−1.7524
		0.03			2.1009	−1.7429	2.1016	−1.7432	2.1038	−1.7433
		0.04			2.1218	−1.7349	2.1225	$\begin{array}{c} -1.7351 \end{array}$	2.1247	−1.7353
			0.02		2.0684	−1.7499	2.0697	−1.7502	2.0741	−1.7509
			0.03		2.0777	−1.7392	2.0797	−1.7395	2.0862	−1.7405
			0.04		2.0868	−1.7296	2.0894	$\begin{array}{c} -1.7300 \end{array}$	2.0981	−1.7313
				0.4	2.2258	−2.1202	2.2271	−2.1215	2.2312	−2.1259
				0.6	2.4441	−2.6358	2.4463	−2.6394	2.4536	−2.6511
				0.8	2.7414	−3.4364	2.7452	−3.4452	2.758	−3.4747

**Table 4:** The effect of different hybridized nanoparticles on Nusselt number (Nu).

	Cu-TiO_2_/H_2_O				Cu-Fe_3_O_4_/H_2_O				Cu-Al_2_O_3_/H_2_O			
$\varphi_{1}\;=\;\varphi_{2}$	$\left|Nu\left(3\right)\right|$	$\left|Nu\left(3.7\right)\right|$	$\left|Nu\left(4.8\right)\right|$	$\left|Nu\left(5.7\right)\right|$	$\left|Nu\left(3\right)\right|$	$\left|Nu\left(3.7\right)\right|$	$\left|Nu\left(4.8\right)\right|$	$\left|Nu\left(5.7\right)\right|$	$\left|Nu\left(3\right)\right|$	$\left|Nu\left(3.7\right)\right|$	$\left|Nu\left(4.8\right)\right|$	$\left|Nu\left(5.7\right)\right|$
1%	1.8586	2.4807	3.2553	3.7454	1.8801	2.4908	3.2586	3.7565.	2.1023	2.7721	3.6048	4.1293
2%	4.4101	4.9099	5.4021	5.6378	4.4251	4.9128	5.4092	5.6484	4.6134	5.1103	5.5781	5.7749
3%	5.5102	5.7584	5.9028	5.8985	5.5122	5.7651	5.914	5.909	5.616	5.8326	5.9358	5.8346
4%	5.9063	5.9308	6.2162	6.3539	5.8959	5.9459	6.4308	6.8651	5.9212	5.9652	6.5901	6.6571

**Table 5:** Different parameter Re (Reynold number), α (wall expansion parameter) *Sc* (Schmidt number) effects on Sherwood number.

			Cu-Al_2_O_3_/H_2_O
Re	α	Sc	$\left|Sh{|}_{\eta =-1}\right|$
−1.5	1	1	0.19442
−1			0.30838
1			1.30681
1.5			1.69612
−1	*−*2		0.62786
	*−*1		0.30838
	1		0.23170
	2		0.05776
	*−*1	2	2.58310
		4	5.6498
		6	8.7222
		8	11.754

**Table 6:** The effect of different physical nondimensional parameters *Re*, *α* (wall expansion), *M* (magneitc parameter), *N* (shape factor), *Ec* (Eckert number), *Pr* (Prandtl number), *Pe* (Peclet number) *θ* (temperature) on heat transfer.

*Re*	*α*	*M*	*N*	Ec	Pr	Pe	$\left|\theta^{\prime }\left(-1\right)\right|$
0.3	0.1	1	3	0.000068	6.2	6.8	1.3807
0.6							2.9588
0.9							4.6361
1.2							5.5035
0.3	0.2						1.1974
	0.3						1.0301
	0.4						0.8791
	0.1	4					1.3661
		7					1.3539
		11					1.3405
		1	3.7				1.3660
			4.8				1.3438
			5.7				1.3265
			3	0.00078			1.3805
				0.00088			1.3803
				0.00098			1.3801
					5.7		1.2891
					6.7		1.4756
					7.7		1.6741
						2.8	1.3817
						5.8	1.3810
						8.8	1.3803

**Table 7:** Magnetic-filed effect in $f^{\prime \prime }\left(\eta \right),\;g^{\prime }\left(\eta \right)$ and $\theta^{\prime }\left(\eta \right)$ at lower wall =−1 $,\;Re=1,\;\varphi_{1}=\varphi_{2}=0.02,\;Ec=0.00068,\;Pr=6.2$.

	**Cu-Al_2_O_3_/H_2_O**		
M	$\;\left|f^{\prime \prime }\left(-1\right)\right|$	$\;\left|g^{\prime }\left(-1\right)\;\right|$	$\;\left|\theta^{\prime }\left(-1\right)\right|$
3	5.5298	3.8675	0.4361
5	5.838	4.2893	1.6694
7	6.1241	4.6544	3.4804
9	6.3914	4.9794	6.0671
11	6.6429	5.2744	8.4787
13	6.8808	5.546	10.7512
15	7.1069	5.7994	12.9101

## Data Availability

All data is available in the manuscript.

## Nomenclature

$B_{o}$	Uniform magnetic field [T]	$F_{\eta }$	Dimensionless radial velocity profile
$G_{\eta }$	Dimensionless tangential velocity profile		
$C_{f}$	Total skin friction coefficient	$\theta_{\eta }$	Dimensionless temperature profile
$C_{p}$	Specific heat at constant pressure	σ	Electrical conductivity [($m^{3}A^{2}$)/kg]
s	Time depended coefficient	υ	Kinematic viscosity [$m^{2}$/s]
M	Magnetic parameter	μ	Dynamic viscosity [$P_{a}$.s]
$P_{r}$	Prandtl number	ρ	Density [kg/$m^{3}]$
r z	Cylindrical coordinates system	$\rho C_{P}$	Volumetric heat capacity [J/(m3 K)]
Re	Reynold number	T	($HN_{fd}$) temperature [K]
u, v, w	velocity component along z axis	$A_{1}$	Dimensionless parameter coefficient
Nu ($HN_{fd}$)	Nusselt number	${\left(\rho c_{p}\right)}_{hnf}$	specific heat capacity for
$\rho_{hnf}$	Density for ($HN_{fd}$)	$\rho_{s1``}$	Density for first solid NP’s
$\rho_{s2}$	Density for second solid NP’s	$k_{hnf}$	Thermal conductivity for ($HN_{fd}$)
$k_{s1}$	Thermal conductivity for first solid fraction	$k_{s2}$	Thermal conductivity for second solid fraction
$k_{mbf}$	Thermal conductivity for shape base fluid	P	pressure
$k_{bf}$	Thermal conductivity for base fluid	$\;\mu_{eff}$	viscosity for effect
$\;\mu_{bf}$	viscosity base fluid	dp	Dimeter of nanoparticles
$dp_{1}$	Dimeter of first nanoparticles	$dp_{2}$	dimeter of 2nd nanoparticles
$\upsilon_{hnf}$	Kinematics viscosity for ($HN_{fd}$)	$Nu_{z}$	Nusselt number
Sc	Schmidt number	Pe	Pectlet number
Ec	Eckert number	N	Size factor
**Subscripts**			
($B_{fd}$)	Base fluid	(NFs)	Nanofluids
(HNFDs)	Hybrid nanofluids		
HNFD1	Cu-TiO_2_ /H_2_O	HNFD2	Ag-Fe_3_O_4_/H_2_O
HNFD3	Cu-Al_2_O_3_ /H_2_O		
**Greek Symbols**			
α	Thermal diffusivity [$m^{2}$/s]	η	Independent similarity variable
φ	Equivalent nanoparticles volume fraction		
$\varphi_{1}$	Equivalent first nanoparticles volume fraction	$\varphi_{2}$	Equivalent first nanoparticles volume fraction
